# Supplementary material for: Diversity of the gut microbiome in three grasshopper species using 16S rRNA and determination of cellulose digestibility
Source: PeerJ. 2020 Nov 5;8:e10194. doi: 10.7717/peerj.10194 (PMC7649011; doi:10.7717/peerj.10194)
Supplement: Supplemental Information 3 [file peerj-08-10194-s003.docx]

**Supplementary Table 1.** Top 10 bacteria genera in terms of average relative abundance in three samples of *Aiolopus tamulus*

| genera | Relative abundance (%) | | | |
| --- | --- | --- | --- | --- |
|  | At1 | At2 | At3 | Mean value |
| *Klebsiella* | \| 51.375 \| 0.744266108 \| 0.961224812 \| \| --- \| --- \| --- \| | 74.427 | 96.122 | 73.975 |
| *Enterococcus* | 10.162 | 1.935 | 0.007 | 4.035 |
| *Enterobacter* | 0.326 | 0.551 | 3.051 | 1.310 |
| *Staphylococcus* | 0.659 | 2.173 | 0.012 | 0.948 |
| *Lactococcus* | 0.164 | 1.738 | 0.000 | 0.634 |
| *Acinetobacter* | 0.442 | 0.365 | 0.012 | 0.273 |
| *Pantoea* | 0.072 | 0.234 | 0.109 | 0.139 |
| *Microbacterium* | 0.020 | 0.051 | 0.000 | 0.024 |
| *Glutamicibacter* | 0.009 | 0.049 | 0.000 | 0.019 |
| *Clavibacter* | 0.015 | 0.028 | 0.002 | 0.015 |
