# Supplementary material for: Diversity of the gut microbiome in three grasshopper species using 16S rRNA and determination of cellulose digestibility
Source: PeerJ. 2020 Nov 5;8:e10194. doi: 10.7717/peerj.10194 (PMC7649011; doi:10.7717/peerj.10194)
Supplement: Supplemental Information 4 [file peerj-08-10194-s004.docx]

**Supplementary Table 2.** Top 10 bacteria genera in terms of average relative abundance in three samples of *Oedaleus decorus asiaticus*

| genera | Relative abundance (%) | | | |
| --- | --- | --- | --- | --- |
|  | Od1 | Od2 | Od3 | Mean value |
| *Klebsiella* | 96.007 | 87.066 | 65.793 | 82.956 |
| *Enterococcus* | 0.000 | 0.005 | 20.085 | 6.696 |
| *Pantoea* | 0.000 | 11.182 | 0.000 | 3.727 |
| *Wolbachia* | 0.229 | 0.357 | 6.880 | 2.486 |
| *Enterobacter* | 0.164 | 1.311 | 0.000 | 1.311 |
| *Lactococcus* | 0.018 | 0.015 | 3.851 | 1.295 |
| *Bromus_tectorum* | 0.084 | 0.274 | 0.000 | 0.119 |
| *Staphylococcus* | 0.005 | 0.023 | 0.026 | 0.018 |
| *Methylobacterium* | 0.005 | 0.026 | 0.009 | 0.013 |
| *Acinetobacter* | 0.007 | 0.020 | 0.011 | 0.012 |
