# Supplementary material for: Diversity of the gut microbiome in three grasshopper species using 16S rRNA and determination of cellulose digestibility
Source: PeerJ. 2020 Nov 5;8:e10194. doi: 10.7717/peerj.10194 (PMC7649011; doi:10.7717/peerj.10194)
Supplement: Supplemental Information 5 [file peerj-08-10194-s005.docx]

**Supplementary Table 3.** Top 10 bacteria genera in terms of average relative abundance in three samples of *Shirakiacris shirakii*

| Genera | Relative abundance (%) | | | |
| --- | --- | --- | --- | --- |
|  | Ss1 | Ss2 | Ss3 | Mean value |
| *Klebsiella* | \| 92.305 \|  \| 0.961224812 \| \| --- \| --- \| --- \| | 94.652 | 87.203 | 91.386 |
| *Lactococcus* | 2.454 | 0.006 | 8.467 | 3.643 |
| *Staphylococcus* | 1.093 | 1.695 | 1.112 | 1.300 |
| *Pseudomonas* | 0.026 | 0.043 | 0.940 | 0.336 |
| *Enterobacter* | 0.172 | 0.099 | 0.678 | 0.316 |
| *Acinetobacter* | 0.459 | 0.431 | 0.007 | 0.299 |
| *Pantoea* | 0.348 | 0.252 | 0.096 | 0.232 |
| *Glutamicibacter* | 0.674 | 0.050 | 0.253 | 0.123 |
| *Clavibacter* | 0.278 | 0.006 | 0.009 | 0.098 |
| *Brachybacterium* | 0.080 | 0.026 | 0.098 | 0.068 |
