# Supplementary figures and images for: Diversity of the gut microbiome in three grasshopper species using 16S rRNA and determination of cellulose digestibility
Source: PeerJ. 2020 Nov 5;8:e10194. doi: 10.7717/peerj.10194 (PMC7649011; doi:10.7717/peerj.10194)

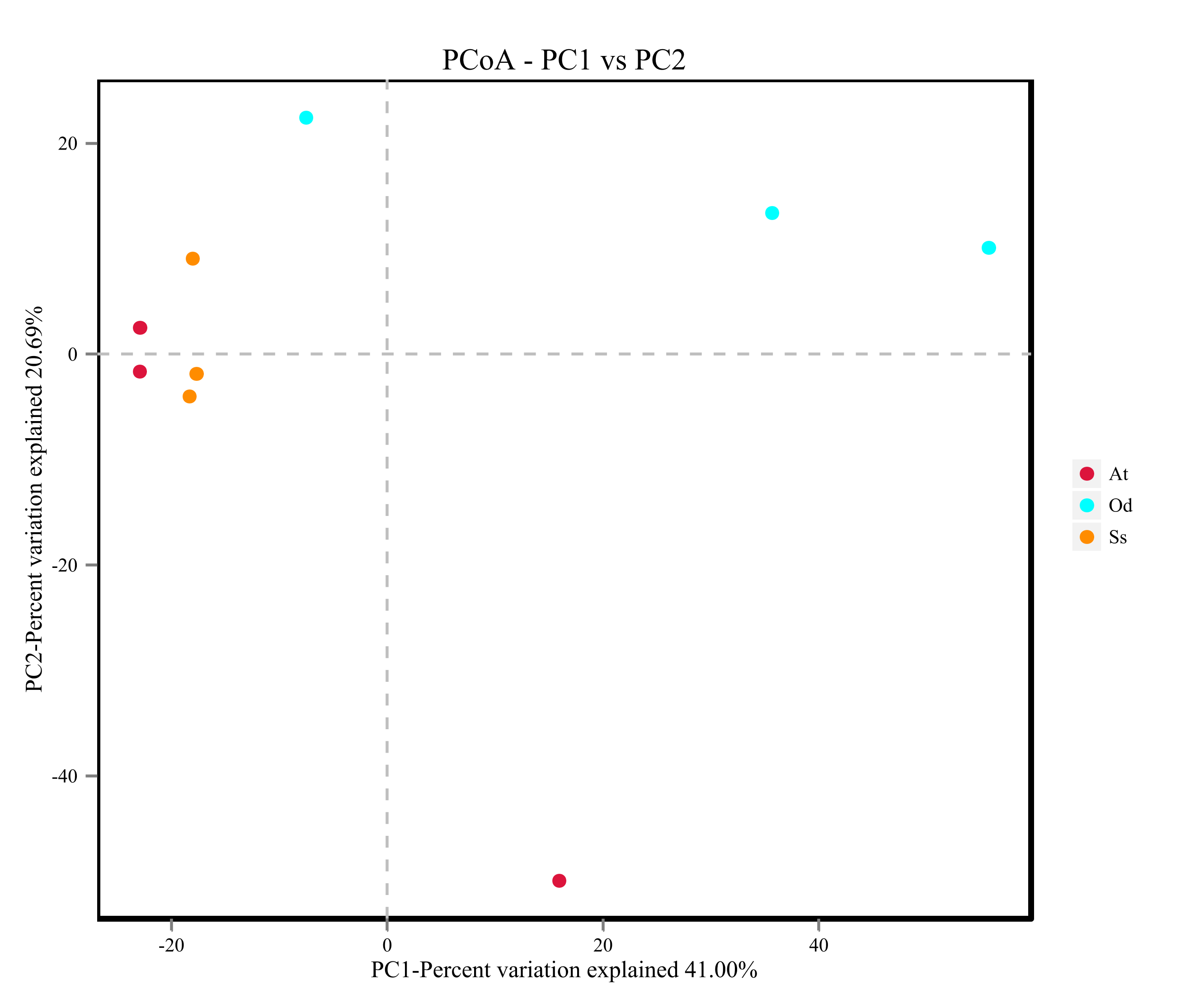

Supplement: Supplemental Information 6 [file peerj-08-10194-s006.png]

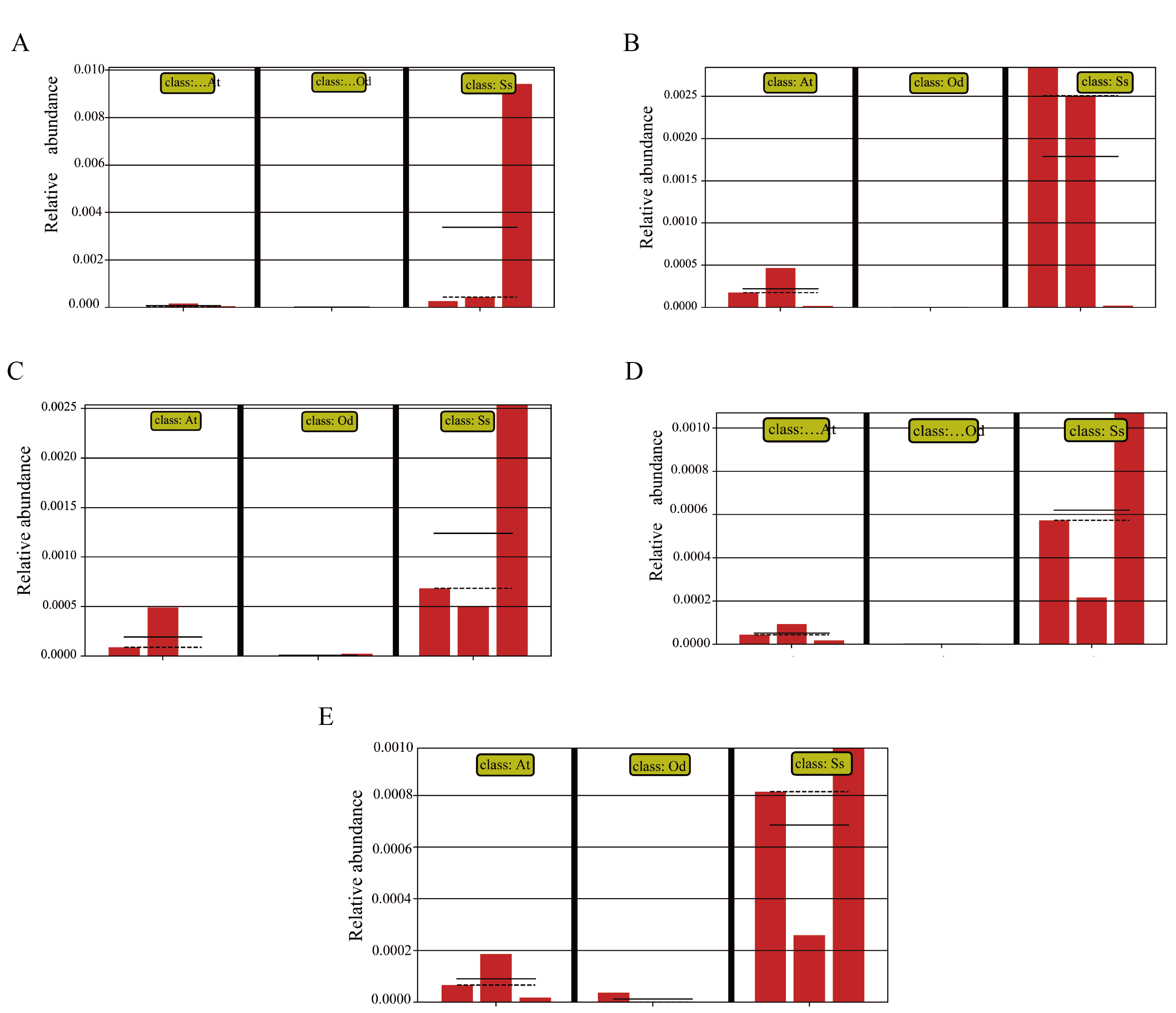

Supplement: Supplemental Information 7 — (A) Pseudomonas. (B) Stenotrophomonas. (C) Glutamicibacter. (D) Corynebacterium. (E) Brachybacterium. [file peerj-08-10194-s007.png]
